# Combinatorial Pharmacogenetic Interactions of Bucindolol and β_1_, α_2C_ Adrenergic Receptor Polymorphisms

**DOI:** 10.1371/journal.pone.0044324

**Published:** 2012-10-10

**Authors:** Christopher M. O'Connor, Mona Fiuzat, Peter E. Carson, Inder S. Anand, Jonathan F. Plehn, Stephen S. Gottlieb, Marc A. Silver, JoAnn Lindenfeld, Alan B. Miller, Michel White, Ryan Walsh, Penny Nelson, Allen Medway, Gordon Davis, Alastair D. Robertson, J. David Port, James Carr, Guinevere A. Murphy, Laura C. Lazzeroni, William T. Abraham, Stephen B. Liggett, Michael R. Bristow

**Affiliations:** 1 Division of Cardiology, Duke University Medical Center/Duke Clinical Research Institute, Durham, North Carolina, United States of America; 2 Division of Cardiology, Department of Veterans Affairs, Washington, District of Columbia, United States of America; 3 Division of Cardiology, Department of Veterans Affairs, Minneapolis, Minnesota, United States of America; 4 National Heart, Lung, and Blood Institute, National Institutes of Health, Washington, District of Columbia, United States of America; 5 Department of Medicine, University of Maryland, Baltimore, Maryland, United States of America; 6 Heart and Vascular Institute, Advocate Christ Medical Center, Oak Lawn, Illinois, United States of America; 7 Division of Cardiology/Cardiovascular Institute, University of Colorado School of Medicine, Aurora, Colorado, United States of America; 8 Division of Cardiology, University of Florida Health Sciences Center, Jacksonville, Florida, United States of America; 9 Research Center, Montreal Heart Institute, Montreal, Quebec, Canada; 10 ARCA biopharma, Broomfield, Colorado, United States of America; 11 Department of Psychiatry and Behavioral United States of America, Sciences and of Pediatrics, Stanford University, Stanford, California, United States of America; 12 Ohio State University, Columbus, Ohio, United States of America; 13 Center for Personalized Medicine and Genomics, University of South Florida, Morsani College of Medicine, Tampa, Florida, United States of America; Leibniz-Institute for Arteriosclerosis Research at the University Muenster, Germany

## Abstract

**Background:**

Pharmacogenetics involves complex interactions of gene products affecting pharmacodynamics and pharmacokinetics, but there is little information on the interaction of multiple genetic modifiers of drug response. Bucindolol is a β-blocker/sympatholytic agent whose efficacy is modulated by polymorphisms in the primary target (β_1_ adrenergic receptor [AR] Arg389 Gly on cardiac myocytes) and a secondary target modifier (α_2C_ AR Ins [wild-type (Wt)] 322–325 deletion [Del] on cardiac adrenergic neurons). The major allele homozygotes and minor allele carriers of each polymorphism are respectively associated with efficacy enhancement and loss, creating the possibility for genotype combination interactions that can be measured by clinical trial methodology.

**Methodology:**

In a 1,040 patient substudy of a bucindolol vs. placebo heart failure clinical trial, we tested the hypothesis that combinations of β_1_389 and α_2C_322–325 polymorphisms are additive for both efficacy enhancement and loss. Additionally, norepinephrine (NE) affinity for β_1_389 AR variants was measured in human explanted left ventricles.

**Principal Findings:**

The combination of β_1_389 Arg+α_2C_322–325 Wt major allele homozygotes (47% of the trial population) was non-additive for efficacy enhancement across six clinical endpoints, with an average efficacy increase of 1.70-fold vs. 2.32-fold in β_1_389 Arg homozygotes+α_2C_322–325 Del minor allele carriers. In contrast, the minor allele carrier combination (13% subset) exhibited additive efficacy loss. These disparate effects are likely due to the higher proportion (42% vs. 8.7%, *P* = 0.009) of high-affinity NE binding sites in β_1_389 Arg vs. Gly ARs, which converts α_2C_Del minor allele-associated NE lowering from a therapeutic liability to a benefit.

**Conclusions:**

On combination, the two sets of AR polymorphisms 1) influenced bucindolol efficacy seemingly unpredictably but consistent with their pharmacologic interactions, and 2) identified subpopulations with enhanced (β_1_389 Arg homozygotes), intermediate (β_1_389 Gly carriers+α_2C_322–325 Wt homozygotes), and no (β_1_389 Gly carriers+α_2C_322–325 Del carriers) efficacy.

## Introduction

Genetic determination of drug action, or pharmacogenetics, involves complex interactions of gene products affecting pharmacodynamics and pharmacokinetics. These interactions are difficult to investigate and quantify, in part because of the uncertainty of the modifier mechanism and the imprecision of measuring drug clinical responses.

There is considerable evidence that genetic variation in bucindolol's primary target, the cardiac β_1_ adrenergic receptor (AR), as well as in a modifier of the signaling mechanism for the primary target, cardiac neuronal norepinephrine (NE) release, modifies the response to this β-blocker/sympatholytic agent and possibly to other anti-adrenergic compounds. Although randomized trials have demonstrated that β-blockers improve survival and clinical outcomes in patients with chronic heart failure (HF) and reduced left ventricular ejection fractions (LVEFs) [1]–[5], trial results vary, with all-cause mortality effect sizes ranging from about 35% [4] to 10% [5]. Differences in the patient populations investigated [6], including geographic origin of study populations [7], may contribute to this variability, but there is also marked response heterogeneity within trials [8]. Genetic variations in ARs [9], [10] could explain the observed high inter-individual variability of β-blocker response [8]. Studies with bucindolol, in development for the indications of chronic HF and atrial fibrillation, have demonstrated response variability dependent on two coding AR polymorphisms that affect signaling: an amino acid position 389 Arg→Gly of the cardiac myocyte located β_1_ AR resulting from a nucleotide position c.1165 C→G in the *ADRB1* gene [11]; and a position 322–325 four amino acid deletion (Del) in the cardiac prejunctional sympathetic nerve terminal α_2C_ AR resulting from a nucleotide position c.964–975 Del in the *ADRA2C* gene [12].

The 389 Arg vs. Gly β_1_ AR variants are pharmacologically distinct. Compared with the β_1_389 Gly AR, the more-common β_1_389 Arg AR has 3-to-4–fold greater signal transduction capacity [11], [13], a higher affinity for agonists [13], [14], and a larger proportion of constitutively active receptors [10], [11]. Bucindolol exerts a selective effect on β_1_389 Arg vs. Gly receptors in part through inverse agonist activity [11], a property that leads to a shift of constitutively active receptors to an inactive state. α_2C_ ARs are localized to cardiac prejunctional nerve terminals, where they mediate inhibition of NE release in a negative feedback loop [15]. The α_2C_ four amino acid Del imparts a loss-of-function phenotype [16] and is also associated with adrenergic dysregulation and an exaggerated sympatholytic response to bucindolol [12]. The presence of both β_1_389 Arg/Arg and α_2C_322–325 Del/Del genotypes appears to synergistically increase the risk of HF in African Americans [9], emphasizing the potential importance of therapeutic interactions between these two AR polymorphisms.

Prejunctional α_2C_ wild-type (Wt) 322–325 Del and postjunctional β_1_Arg389 Gly genetic variants are positioned in-series in the cardiac adrenergic neuroeffector pathway, and their potential interaction provides an opportunity to investigate the genetic complexity [17], [18] of drug response. Most treatment effects are likely determined by the interplay of multiple genes [17], and the existence of two drug-response modifying polymorphisms in-series with a signaling pathway that is a major determinant of HF disease progression provides a unique model system for investigating complex pharmacogenetic interactions. Given the pharmacologic and clinical importance of these polymorphisms, in a 1,040 patient substudy of the Beta Blocker Evaluation of Survival Trial (BEST), we investigated their combined influence on major cardiovascular event responses to bucindolol by testing the primary hypothesis that the efficacy-modifying effects of each polymorphism would be additive and more pharmacogenetically informative when genotypes are combined. To provide adequate precision of detection of pharmacogenetic effects, we investigated effects on six BEST Endpoint Committee-adjudicated heart failure clinical outcomes, two of which (all-cause mortality and all-cause mortality or cardiac transplantation) were the primary endpoints of the substudy. In addition, when results were obtained that were inconsistent with the primary hypothesis, in experiments performed in left ventricular (LV) membrane preparations expressing β_1_ ARs, we tested the subhypothesis that marked differences in NE affinity accounted for the disparate results.

## Methods

The 2,708-patient BEST trial [5], sponsored by the Department of Veterans Affairs and the National Heart, Lung, and Blood Institute, measured how bucindolol affected clinical endpoints in advanced chronic HF patients. The primary endpoint of BEST was all-cause mortality, which when analyzed according to the regulatory statistical analysis plan that included covariate adjustment for randomization stratifying variable and censoring for cardiac transplantation, yielded a hazard ratio (HR) and 95% confidence interval (CI) of 0.87 (0.76–1.00), *P* = 0.053. BEST contained a 1,040-patient DNA bank, which could be accessed by submission and successful peer review of a substudy protocol [11], [12]. Pharmacogenetic data presented here are from the DNA Oversight Committee-approved substudy “Pharmacogenomics of Beta-Adrenergic Receptor Polymorphisms and Response to Beta-Blockers in Heart Failure,” which was submitted prior to the trial's ending while patients were still being enrolled. The substudy tested the hypothesis that six previously identified AR variants [11], [12], of which five occur at a frequency adequate for hypothesis testing, could predict β-blocker response heterogeneity. This protocol included a provision for examining the effects of combinations of polymorphisms when positive findings were obtained for individual variants; an addendum to the protocol submitted prior to completion of the genetic analysis prospectively identified the potential importance of combinatorial interactions of β_1_389 and α_2C_322–325 polymorphisms.

The protocol-defined primary statistical method was Cox regression analysis [11], [12], yielding HRs and 95% CIs within genetic subgroups by treatment type or within treatment group by genotype. Because of racial differences in the distribution of AR polymorphisms, Cox models were covariate-adjusted for race and the three other BEST randomization stratification variables (+/− ischemic cardiomyopathy etiology of HF, LVEF, and sex). Tests for interaction were run on treatment group/genotype comparisons. HRs from Cox regression analyses or relative change ratios (RCRs) from HF hospitalization (HFH) days/patient data were transformed and normalized to the results in the entire DNA substudy cohort by the relative effect size (RES) method [12] of RES = Ln (HR or RCR genotype group)/Ln (HR or RCR DNA substudy cohort). Differential efficacy in percentage was calculated by adding the RES interval above unity to the amount below, and multiplying by 100. Results are presented as intention-to-treat analyses from the time of randomization and for the substudy's two primary clinical endpoints (times to all-cause mortality and all-cause mortality or cardiac transplantation), four secondary clinical endpoints (three of which were BEST parent protocol secondary endpoints, and the fourth, time to cardiovascular hospitalization, was requested post hoc by the U.S. Food and Drug Administration), and a negative control endpoint (time to non-cardiovascular hospitalization). All four of these efficacy secondary endpoints had a *P*<0.050 in the entire cohort. For efficacy analyses with genetic subgroups in this exploratory study, *P* values<0.050 in a two-tailed distribution were considered of interest and *P* values<0.010 were considered statistically significant based on a prespecified multiple comparison adjustment described for monotypes in the substudy grant application statistical section. A comparable multiple comparison adjustment for the four combination genotypes investigated in this study would yield a critical value of 0.0125.

High-affinity “agonist” binding of L-NE to β_1_ ARs was determined in non-failing human left ventricles obtained from unused organ donors as previously described [19].

The BEST trial was conducted according to Declaration of Helsinki principles. All participating patients gave informed written consent for both the parent protocol and the DNA substudy. Non-failing human hearts were provided by Donor Alliance, the Colorado-Wyoming organ procurement organization, which obtained written consent for research use of tissue from donor family members.

## Results

### Study population

The patient population was classified by the four possible β_1_389 Arg/Gly and α_2C_Wt/Del combinations of major allele homozygote and minor allele carrier [11], [12] genotype combination groups 1–4, ordered by the number of major allele homozygous monotypes (Table 1).

**Table 1:** Baseline characteristics by genotype combination groups.

Characteristic	Group 1^A^ (n = 420)	Group 2^B^ (n = 73)	Group 3^C^ (n = 413)	Group 4^D^ (n = 134)
β_1_389 genotype	Arg/Arg	Arg/Arg	Gly Carrier	Gly Carrier
α_2C_322–325 genotype	Wt/Wt	Del Carrier	Wt/Wt	_Del Carrier_
Age, y^E^	60.7±11.3	56.9±13.7	61.0±12.4	58.9±12.7
Male sex, n (%)	339 (81)	54 (74)	332 (80)	100 (75)
African American,^E^ n (%)	19 (5)^F^	47 (64)	50 (12)^F^	91 (68)
CHF duration, mo	46.6±49.6	55.9±56.8	41.4±41.9	48.1±48.8
NYHA class, n (%)				
III	397 (95)	68 (93)	373 (90)	122 (91)
IV	23 (5)	5 (7)	40 (10)	12 (9)
Systolic BP, mmHg^E^	117±17.8	123±19.8	118±17.2	120±18.7
HF etiology, n (%)				
Ischemic^G^	242 (58)	41 (56)	258 (62)^H^	65 (49)
Non-ischemic^G^	178 (42)	32 (44)	155 (38)^H^	69 (51)
LVEF, %	23.3±7.1	23.6±7.1	24.0±7.0	23.3±7.1
Diabetes, n (%)	148 (35)	30 (41)	133 (32)	51 (38)
History of hypertension,^G^ n (%)	202 (48)	53 (73)^I^	228 (55)^H^^,^ J	97 (72)^I^
Concomitant medications, n (%)				
ACEIs	382 (91)	69 (95)	381 (92)	128 (96)
Diuretics	387 (92)	67 (92)	388 (94)	125 (93)
Digoxin	384 (91)	63 (86)	372 (90)	117 (87)
NE, pg/mL	472±279 (n = 352)	517±349 (n = 59)	488±250 (n = 363)	523±354 (n = 101)

Data presented as means ± standard deviations, unless otherwise noted.

Abbreviations: ACEI, angiotensin-converting enzyme inhibitor; BP, blood pressure; CHF, chronic heart failure; HF, heart failure; LVEF, left ventricular ejection fraction; NE, norepinephrine; NYHA, New York Heart Association.

A β**_1_**389 Arg/Arg+α**_2C_**_Wt/_Wt.

B β**_1_**389 Arg/Arg+α**_2C_**_Del_ carrier.

C β**_1_**389 Gly carrier+α_2c Wt/_Wt.

D β**_1_**389 Gly carrier+α**_2C_**_Del_ carrier.

E *P*<0.05 by ANOVA (ANalysis Of Variance).

F *P*<0.0083 by Bonferroni for all pair wise comparisons.

G *P*<0.05 by Chi-square test.

H *P*<0.0083 by Bonferroni vs. Group 4.

I *P*<0.0083 by Bonferroni vs. Group 1.

J *P*<0.0083 by Bonferroni vs. Group 2.

Baseline characteristics of the genotype combination groups are given in Table 1. The most striking difference between genotype combinations is race where, as expected [9], there was a greater percentage of African American patients in the two groups (2 and 4) containing α_2C_322–325 Del alleles. Other differences among genotype combinations, such as more non-ischemic cardiomyopathy etiology in Group 3 vs. Group 4 and a greater proportion of patients with a history of hypertension in groups 2 and 4, are likely due to the racial imbalance. The small, clinically insignificant ANOVA (ANalysis Of VAriance between groups) differences in age and systolic blood pressure are not statistically significant after a multiple comparison adjustment.

### Outcomes by β_1_389 Arg/Gly and α_2C_322–325 Wt/Del AR genotype combinations

Table 2 gives the HRs or RCRs for the various clinical efficacy endpoints. For Groups 1 (β_1_389 Arg/Arg+α_2C_322–325 Wt/Wt) and 2 (β_1_389 Arg/Arg+α_2C_322–325 Del carrier), the six HRs and RCRs overlap with respective average effect sizes of 40% and 49%, with both groups exceeding the average effect size of 26% in the all-genotype 1,040-patient DNA substudy cohort. RES, which normalizes the effect size to that in the DNA substudy cohort, yielded average values of 1.70 and 2.32, respectively, in Groups 1 and 2. This means efficacy was increased by an average of 70% (Group 1) or 132% (Group 2) relative to the parent population containing all genotypes. However, the RES range for Groups 1 and 2 overlapped (Table 2; 1.48–2.14 for Group 1; 1.49–3.29 for Group 2). Because of the small numbers of patients and events in Group 2, only Group 1 HRs or RCRs achieved *P* values<0.010 (heart failure progression [HFP] and HFH days/patient) or <0.05 (all endpoints but all-cause mortality, *P* = 0.099).

**Table 2:** Hazard ratios or relative change ratios for bucindolol/placebo (95% confidence intervals), number of events, and log-rank *P* values for clinical endpoints and norepinephrine change by genotype combination groups.

Endpoint (no. events in DNA substudy cohort)^A^	DNA substudy cohort^B^	Group 1^C^	Group 2^D^	Group 1/2^E^	Group 3^F^	Group 4^G^
	n = 1040 (525P, 515B)	n = 420 (207P, 213B)	n = 73 (29P, 44B)	n = 493 (236P, 257B)	n = 413 (214P, 199B)	n = 134 (75P, 59B)
ACM						
HR/RCR (95% CI)	0.77 (0.58–1.03)	0.66 (0.39–1.09)	0.50 (0.12–2.05)	0.62^H^ (0.39–0.99)	0.75^H^ (0.48–1.17)	1.04^H^ (0.43–2.54)
No. events	189	67	13	80	85	24
Log-rank *P* values	0.077	0.099	0.33	0.042	0.21	0.93
ACM/transplant						
HR/RCR (95% CI)	0.74 (0.56–0.98)	0.59 (0.37–0.96)	0.50 (0.12–2.05)	0.57^I^ (0.36–0.89)	0.76^I^ (0.50–1.16)	1.04^I^ (0.43–2.54)
No. events	207	75	13	88	94	25
Log-rank *P* values	0.035	0.031	0.33	0.012	0.20	0.93
CVM						
HR/RCR (95% CI)	0.66 (0.48–0.91)	0.54 (0.31–0.97)	0.40 (0.08–2.12)	0.52^I^ (0.31–0.88)	0.60^I^ (0.36–0.97)	1.11^I^ (0.45–2.78)
No. events	159	54	10	64	73	22
Log-rank *P* values	0.011	0.035	0.27	0.014	0.036	0.82
HFP						
HR/RCR (95% CI)	0.76 (0.62–0.92)	0.65 (0.47–0.89)	0.58 (0.27–1.25)	0.66^H^ (0.49–0.88)	0.80^H^ (0.60–1.08)	0.99^H^ (0.53–1.84)
No. events	436	165	32	197	188	51
Log-rank *P* values	0.004	0.007	0.16	0.005	0.14	0.96
HFH days/patient^J^						
HR/RCR (95% CI)	0.71 (0.47–0.95)	0.48 (0.18–0.78)	0.60 (−0.11–1.30)	0.52^I^ (0.24–0.80)	0.83^I^ (0.47–1.20)	1.19^I^ (−0.17–2.55)
No. days	5805	5805	343	2632	2281	892
*P* values	0.042	0.009	0.38	0.009	0.41	0.76
CVH						
HR/RCR (95% CI)	0.79 (0.66–0.96)	0.68 (0.50–0.93)	0.46 (0.21–1.02)	0.64^H^ (0.48–0.86)	0.91^H^ (0.68–1.22)	0.96^H^ (0.53–1.76)
No. events	447	171	33	204	190	53
Log-rank *P* values	0.016	0.016	0.051	0.002	0.53	0.90
Average effect size^K^, %	26	40	49	41	22	−5.5
Average RES^L^ (range)	1.00	1.70 (1.48–2.14)	2.32 (1.49–3.29)	1.76 (1.51–1.91)	0.83 (0.40–1.22)	−0.14 (−0.51–0.17)
Differential efficacy^M^, %	–	184	246	190	97	–
Non-CVH						
HR/RCR (95% CI)	0.99 (0.80–1.22)	0.87 (0.62–1.23)	1.59 (0.66–3.87)	0.94 (0.69–1.28)	1.11 (0.80–1.55)	0.99 (0.48–2.01)
No. events	367	145	28	173	153	41
Log-rank *P* values	0.94	0.43	0.30	0.69	0.53	0.97
ΔNE at 3 mo, pg/mL						
Placebo	17±15	−4±19	38±110P	1.0±21	28±21	31±60
Bucindolol	−66±15	−50±19	−120±82	−62±20	−50±22	−145±64
Log-rank *P* values	<0.001	0.091	0.27	0.027	0.011	0.0496
B group - P group as net change relative to placebo	−83	−46	−158	−63	−78	−176

Abbreviations: ACM, all-cause mortality; B, bucindolol; CI, confidence interval; CVH, cardiovascular hospitalization; CVM, cardiovascular mortality; HFH, heart failure hospitalization; HFP, heart failure progression (composite of heart failure death, cardiac transplantation, heart failure hospitalization, or an emergency department visit for treatment of heart failure involving administration of intravenous heart failure medication); HR, hazard ratio; P, placebo; NE, norepinephrine; RCR, relative change ratio; RES, relative effect size.

A Number of events presented are using the unadjusted analysis, which differs slightly from covariate-adjusted because adjusted analyses are transplant-censored.

B All genotypes.

C β_1_389 Arg/Arg+α_2C Wt/_Wt.

D β_1_389 Arg/Arg+α_2C Del_ carrier.

E β_1_389 Arg/Arg+any α_2C_.

F β_1_389 Gly carrier+α_2C Wt/_Wt.

G β_1_389 Gly carrier+α_2C Del_ carrier.

H Interaction *P* value≤0.20, >0.10.

I Interaction *P* value≤0.10, >0.05.

J Relative change ratio.

K [1−HR or RCR]×100.

L RES = Ln HR (genotype group)/Ln (DNA substudy cohort).

M See Methods.

The HR/RCR and RES data for Group 2 therefore indicate that when combined with the β_1_389 major allele (Arg) homozygous monotype, the α_2C_322–325 Del variant does not exert any negative influence on bucindolol treatment effects despite Group 2 exhibiting a typical α_2C_322–325 Del variant-associated large decrease in NE (by 158 pg/mL compared with placebo), a degree of sympatholysis previously identified as compromising efficacy [12], [20]. In contrast, Group 1, which contains the Wt/Wt version of the α_2C_ AR gene, exhibited only the expected mild NE lowering (average of 46 pg/mL compared with placebo) in bucindolol-treated patients (Table 2), a degree of sympatholysis that has been associated with increased efficacy [12], [20]. Therefore, there is no evidence that the presence of the α_2C_ Del allele or degree of sympatholysis adversely influences outcomes when the β_1_389 polymorphism is Arg/Arg. Because the RES values for Groups 1 and 2 overlap, Group 1/2 was created as a combination genotype consisting of β_1_389 Arg/Arg+either α_2C_322–325 variant. Hazard ratios, RCRs, and *P* values in Group 1/2 are similar to those of Group 1 (Table 2), with respective average effect and relative effect sizes of 41% and 1.76 for Group 1/2, and 40% and 1.70 for Group 1. For Group 1/2, all *P* values for efficacy endpoints are <0.050; three are <0.010; and four, including the coprimary endpoint of ACM/transplant, are <0.0125 (the critical value for genotype combinations adjusted for multiple comparison).

As shown in Table 2, Group 3 (β_1_389 Gly carrier+α_2C_322–325 Wt/Wt) exhibited HRs/RCRs that were generally greater (less efficacy) than in Group 1 or Group 1/2 and similar to those in the 1,040-patient DNA substudy cohort. Despite a sample size similar to Group 1, only cardiovascular mortality had a *P*<0.05, and no endpoint had a *P*<0.010. The average effect size for Group 3 was 22%, compared with 26% in the entire DNA substudy, and the average RES was 0.83. The upper bound of the Group 3 RES range, 1.22, did not overlap with either the Group 1 (1.48), Group 2 (1.49) or Group 1/2 (1.51) lower bound of the RES range. Strikingly, Group 4 (β_1_389 Gly carrier+α_2C_322–325 Del carrier) exhibited HRs/RCRs of ∼1.0 with an average effect size of −5.5%, or no evidence of any efficacy. The average RES in Group 4 was −0.14 (range, −0.51–0.17), indicating higher event rates in the bucindolol group vs. the placebo group. This is comparable to respective average RES values of 0.40 (range, 0.22–0.60) and 0.22 (range −0.03–0.79) in the monotypes β_1_389 Gly carrier and α_2C_ Del carrier, respectively (data not shown). The upper bound of the Group 4 RES range (0.17) does not overlap with the lower bound of the Group 3 RES range (0.40). Thus, when β_1_389 Gly carriers are combined with α_2C_322–325 Del carriers, an additive loss of efficacy occurs, compared with Groups 2 or 3, which contain only one monotype minor allele carrier. The reduction in NE at three months in Group 4 was expectedly large because of the presence of α_2C_322–325 Del alleles, and this degree of sympatholysis [12], [20] in patients with hypofunctional β_1_389 Gly receptors may have been the reason for efficacy loss.

Table 2 also gives the differential efficacy calculation [12], which is an important pharmacogenetic measure that expresses the maximal degree of efficacy separation of genetically defined groups. Compared with Group 4, Groups 1/2 and 3 had differential efficacies of 190% and 97%, respectively. These are comparable to differential efficacies of 136% for Group 1/2 vs. β_1_389 Gly carrier (average RES for the six clinical efficacy endpoints, 0.40) and 96% for α_2C_322–325 Wt/Wt (average RES,1.18) vs. Del carrier (average RES, 0.22). Thus, the differential efficacy gained by comparing Group 1/2 with Group 4 vs. the β_1_389 Gly carrier monotype is calculated as follows: 190%–136% = 54%. The advantage vs. the α_2C_322–325 Del carrier monotype is calculated as follows: 190%–96% = 94%.

Interaction tests with Groups 1/2, 3, and 4 as a continuous ordinal variable were *P* = 0.13 and 0.093, respectively, for all-cause mortality and all-cause mortality or transplant, 0.073 for HFH days/patient, and <0.20 for two of the three other efficacy endpoints. In contrast to the efficacy endpoints, there was no evidence of an effect of bucindolol or genotype combination on time to non-cardiovascular hospitalization, with an average HR/RCR of 0.99 in the entire cohort and a range of 0.87–1.59 in genotype groups.

### Time-to-event curves for β_1_389 Arg/Gly and α_2C_322–325 Wt/Del combination genotypes

**Figure 1:**
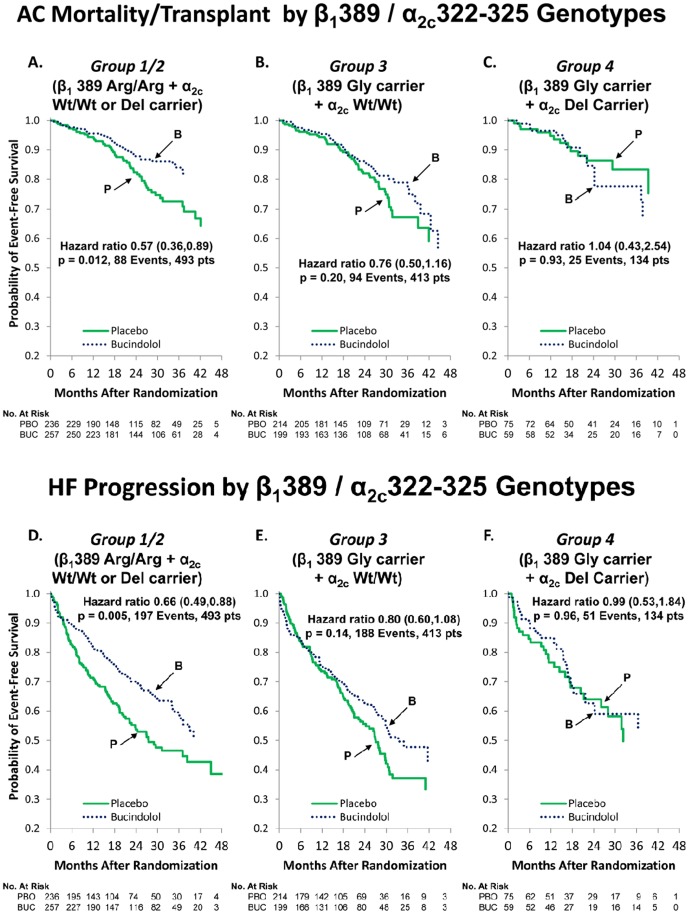
Time to all-cause mortality or cardiac transplantation for Group 1/2 (A), Group 3 (B), and Group 4 (C), and time to heart failure progression (combination endpoint of heart failure mortality, cardiac transplantation, heart failure hospitalization, or emergency department care that includes intravenous therapy not requiring hospitalization) for Group 1/2 (D), Group 3 (E), and Group 4 (F). Abbreviations: AC, all-cause; BUC, bucindolol; Del, deletion; HF, heart failure; PBO, placebo.

Figures 1A, 1B, and 1C give Kaplan-Meier curves for the primary endpoint of all-cause mortality or cardiac transplantation (ACM/Tx) for the efficacy-enhanced Group 1/2 (1A), the intermediate-efficacy Group 3 (1B), and the loss-of-efficacy Group 4 (1C) genotype combinations. Figures 1D, 1E, and 1F give the Kaplan-Meier curves for the combined endpoint of HFP in these same genotype groups. For both clinical endpoints, the pattern is substantial, statistically significant separation of the bucindolol and placebo curves in Group 1/2, moderate separation but non-significant *P* values in Group 3, and no curve separation (Figure 1F) or even curve crossover (Figure 1C) in Group 4. The HRs (95% CIs) for the pharmacogenetic groups are given in Table 2 and can be compared with those for the 2,708-patient entire cohort (ACM/Tx HR, 0.86; 95% CI, 0.75–0.98; *P* = 0.021) (HFP HR, 0.80; 95% CI, 0.72–0.89; *P*<0.0001) or the similar values for the 1,040-patient DNA substudy (Table 2). The Group 1/2 HRs of 0.57 (95% CI, 0.36–0.89; *P* = 0.012) for ACM/Tx and 0.66 (95% CI, 0.49–0.88; *P* = 0.005) for HFP (Table 2) are substantially less than the respective HRs for the entire or DNA substudy cohorts, with *P* values that are at or below the prespecified statistical analysis plan critical values.

### High-affinity agonist binding by L-NE to Arg or Gly β_1_389 ARs

**Figure 2:**
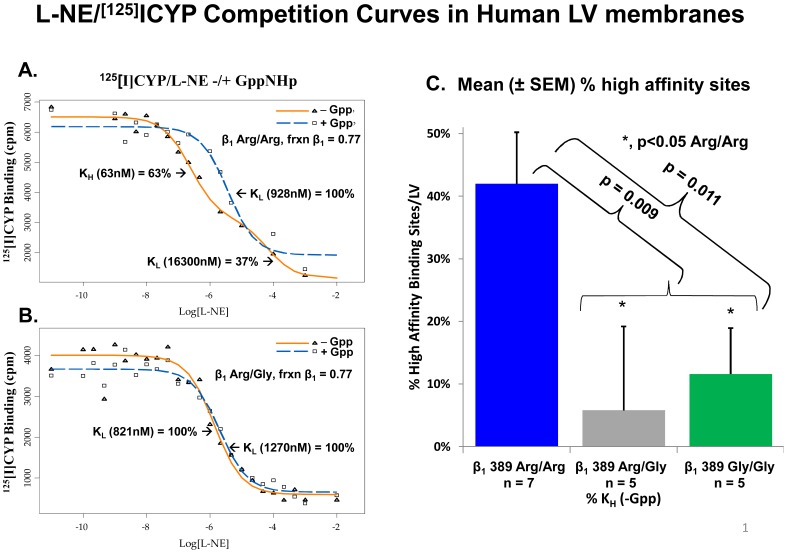
Representative competition curves between 50 pM ^125^[I]CYP and L-NE at increasing concentrations, in the absence and presence of 30 µM Gpp(NH)p in membranes from a non-failing human left ventricle with 77% β_1_ AR that was β_1_389 Arg/Arg genotype (A) and in membranes from a non-failing human heart with 77% β_1_ AR that was β_1_389 Arg/Gly genotype (B); mean±SEM (%) of high-affinity L-NE binding sites identified in seven β_1_389 Arg/Arg, five β_1_389 Arg/Gly, and five β_1_389 Gly/Gly left ventricles (C). Abbreviations: AR, adrenergic receptor; CYP, cyanopindolol; Gpp(NH)p, non-hydrolyzable guanine nucleotide; K_H_, dissociation constant; K_L_, low-affinity binding constant; L-NE, L-norepinephrine; SEM, standard error measurement.

To investigate the affinity of NE for β_1_ ARs of various genotypes, we measured high-affinity agonist binding in membranes prepared from 17 human non-failing LV membranes that contained at least 75% β_1_ ARs (mean, 80.0±5.8%). Figure 2 gives representative competition curves for L-NE and ^125^[I]CYP (cyanopindolol) in a β_1_389 Arg/Arg (Figure 2A) or a β_1_389 heterozygote preparation (Figure 2B). In the β_1_389 Arg/Arg preparation, there is high-affinity displacement of ^125^[I]CYP by L-NE, with a dissociation constant (K_H_) of 63 nM. Computer modeling yielded a two-site fit, with the high-/low-affinity percentages of β_1_ ARs estimated to be 63/37. When incubated with the non-hydrolyzable guanine nucleotide Gpp(NH)p, which uncouples high-affinity agonist binding, only low-affinity (K_L_ 928 nM) receptors are identified. In contrast, in the competition curve from a left ventricle genotyped as β_1_389 Arg/Gly, there is no evidence of any high-affinity L-NE binding. In all, 6/7 Arg/Arg; 1/5 Arg/Gly; and 2/5 Gly/Gly left ventricles exhibited two-site fits in the absence of Gpp(NH)p, with an average K_H_ of 74.9±87.0 nM. The low-affinity binding constant (K_L_) in all 17 left ventricles averaged 7,254±12,250 nM. Figure 2C also gives the mean percentages of high-affinity L-NE β_1_ ARs in the three possible genotypes. Both heterozygotes (5.8%) and β_1_389 Gly homozygotes (11.6%) have much lower percentages of high-affinity L-NE binding sites than Arg homozygotes (42.0%, ANOVA *P* = 0.011; *P* = 0.009 for Arg/Arg vs. Gly carriers).

## Discussion

Based on an analysis of three clinical endpoints, we have previously reported [11] that the the β-blocker/sympatholytic agent bucindolol exhibits enhanced clinical efficacy in ∼50% of the population that is homozygous for an Arg allele at position 389 of the β_1_ AR, compared with patients with a Gly allele at this position. Two of these endpoints (time to all-cause mortality and all-cause mortality/HFH) were also measured in the current study, and the other (time to first HFH) was the major component of time to HF progression in the current report. In another study [12] based on five clinical endpoints, all of which are included in the current report, we found that the ∼80% of patients with an Ins (Wt) at amino acids 322–325 of the α_2C_ AR had therapeutic responses that were consistently better than in patients with a deletion of amino acids 322–325. This α_2C_ AR 322–325 polymorphism-associated differential clinical response was found to be related to dysregulation of NE release in patients with Del genotypes, who had exaggerated sympatholytic responses to bucindolol that obviated efficacy. The major allele homozygotes of these two AR polymorphisms are therefore associated with enhanced response. In the case of β_1_389, this response is enhanced to a degree that tends to be greater than the response to standard β-blockers in all genotypes. In the case of α_2C_322–325, this response is enhanced to a degree comparable to standard β-blockers in all genotypes. Thus, the question arises as to whether the combination of two major allele genotypes would yield an even greater therapeutic response than in either monotype alone. This provides an opportunity to assess the interactive effects of two genetic variants that are intimately related to disease pathophysiology as well as drug response, a rare opportunity in pharmacogenetics. To test the hypothesis that the effects of each of these AR polymorphisms would be additive for HF clinical response, we investigated the effects of bucindolol vs. placebo on six clinical endpoints that included the three previously measured for β_1_389 Arg/Gly effects [11] and the five for α_2C_322–325 Wt/Del effects [12]. We added an additional adjudicated efficacy endpoint, cardiovascular hospitalization, and also included a negative control, non-efficacy endpoint of non-cardiovascular hospitalization.

Surprisingly, no additional therapeutic benefit of bucindolol was observed in the major allele homozygote combination of β_1_389 Arg/Arg+α_2C_322–325 Wt/Wt. That is, efficacy (as measured by the RES method) [12] across the six efficacy endpoints was enhanced by an average of 1.70-fold in the major allele homozygotes combination genotype, compared with 2.32-fold in the combination genotype of β_1_389 Arg/Arg+α_2C_322–325 Del carrier. The non-efficacy, non-cardiovascular hospitalization endpoint did not exhibit pharmacogenetic enhancement or decrement. In contrast, for carriers of β_1_389 and α_2C_322–325 minor alleles (Group 4) as a combination genotype, there was additive diminished efficacy to the point of complete efficacy loss (RES = −0.14). This indicates an average RES for which the bucindolol treatment effect is 14% worse than placebo and worse than in patients with the β_1_389 Gly carrier or α_2C_ Del carrier monotype, for which the respective RES values indicated efficacies 40% or 22% better than placebo. In Group 4, the further decrement in efficacy compared with the β_1_389 Gly carrier monotype was due to the removal of the β_1_389 Gly carrier+α_2C_ Wt/Wt combined genotype as Group 3. Compared with the α_2C_ Del carrier monotype, this was due to the removal of the β_1_389 Arg/Arg+α_2C_ Del carrier combined genotype to constitute Group 2. Thus the hypothesis that combining predictive individual monotypes into combined genotypes improves pharmacogenetic targeting was supported only for the minor alleles.

The differences between minor and major alleles in pharmacogenetic combination are likely due to the pharmacologic differences in β_1_389 Arg vs. Gly receptors. Compared with its Gly counterpart, the β_1_389 Arg receptor has higher signal transduction capacity [11], [13], more receptors in a constitutively active state [10], [11], and higher-agonist affinity [13], [14]. We demonstrated that high-affinity agonist binding to NE does not extend to heterozygotes, as Gly β_1_389 ARs appear to exert a dominant negative effect on NE high-affinity binding as well as on bucindolol's clinical efficacy. Therefore, in advanced HF patients, the 389 Arg version of the β_1_ AR is much better-equipped to support cardiac function in the face of marked NE lowering, where its higher-agonist binding affinity allows it to better utilize low levels of adrenergic activity. In contrast, the hypofunctioning, lower-NE affinity β_1_389 Gly version of the β_1_ AR needs higher NE levels to support the failing heart, and in the presence of the α_2C_322–325 Del-associated marked sympatholysis, likely cannot adequately support cardiac function, leading to an increase in mortality and hospitalizations that cancels bucindolol efficacy. These relationships likely explain the marked differences in bucindolol's clinical efficacy between Group 2 (has only β_1_389 Arg receptors) and Group 4 (contains ≥50% β_1_389 Gly receptors). Moreover, because of its much higher affinity for NE, the β_1_389 Arg receptor is an NE receptor, whereas the β_1_389 Gly variant is not, having an NE affinity that is similar to the β_2_ AR. The sympatholytic effects of bucindolol therefore preferentially inhibit β_1_389 Arg signalling, providing a basis for the selective clinical effects of bucindolol in β_1_389 Arg/Arg genotypes vs. any Gly-containing genotype.

The β_1_389 and α_2C_322–325 combinations of genetic biomarkers conferred a nearly two-fold difference in averaged efficacy (a differential efficacy of 190%) between the efficacy-enhanced (β_1_389 Arg/Arg) genotype Group 1/2 and the loss-of-efficacy (β_1_389 Gly carrier, α_2C_322–325 Del carrier) genotype Group 4. The goal of pharmacogenetic targeting is to identify subgroups with large differences in treatment efficacy, or “outliers” [21], so that the more responsive group can be offered the likelihood of benefit that is better than that in the general population, and the less-responsive group can avoid treatment exposure. In this regard, the use of β_1_389 and α_2C_ combination genotypes yielded numerically greater degrees of high-low response differential efficacy compared with β_1_389 or α_2C_ monotypes, by respective absolute amounts of 54% and 94%. Based on the above and non-overlap of RES ranges, combinations of β_1_389 Arg/Gly and α_2C_322–325 genotypes therefore identified a 47% subpopulation (Group 1/2 [β_1_389 Arg/Arg+any α_2C_]) with a bucindolol-enhanced clinical response profile compared with the parent population that generally exceeds effect sizes associated with other β-blockers in a variety of HF populations [1]–[8], a 40% subpopulation (Group 3 [β_1_389 Gly carriers+α_2C_322–325 Wt/Wt]) with clinical responses similar to that in the parent population of all genotypes, and a small but non-trivial 13% subpopulation with complete loss of efficacy that should not be treated with bucindolol.

Although tests for interaction between β_1_389 and α_2C_ genotype combinations and treatment effects did not achieve statistical significance, two *P* values were <0.10 and most were <0.20. However, interaction tests, commonly used to assess heterogeneity of subgroups within a clinical trial population without regard to any particular mechanistic interaction with the tested treatment, have limited statistical power to detect pharmacogenetic efficacy differences that can be expected to be small, unidirectional, and present in limited sample sizes. The demonstration of statistically significant, robust treatment effects across multiple relevant clinical endpoints in one pharmacogenetic subset but not its allelic counterpart, especially when supported by biologic plausibility, achieves the goal of identifying a subset of patients highly likely to respond favorably to a treatment. The β_1_389 Arg/Arg genotype meets these criteria for a favorable response pharmacogenetic subgroup.

### Limitations

There are some limitations to this study. First, although the substudy was prospectively designed and hypothesis-driven, the pharmacogenetic data were generated and analyzed after the trial's main results were analyzed and published [5]. However, the investigators generating the pharmacogenetic data remained blinded to the treatment code and to clinical outcomes throughout. Second, approximately two-thirds of the patients were enrolled into the DNA substudy after being randomized into the parent trial. This “late entry” phenomenon has been extensively analyzed, by both L-truncation [12] and, most recently, propensity score statistical methods (unpublished observations). The effect of late entry into the DNA substudy is only to lower event rates for all clinical endpoints, without affecting genotype-specific treatment effects.

### Conclusions

The combinatorial interaction of two sets of AR polymorphisms that influence bucindolol's drug action resulted in unanticipated effects on HF clinical responses, non-additivity in efficacy enhancement for the major allele homozygotes, and additive effects for minor allele carrier-associated efficacy loss. An explanation for these disparate results was provided by the effects of the α_2C_322–325 minor (Del) allele on facilitating bucindolol's NE-lowering properties, where excessive NE lowering abolished efficacy when the β_1_389 Gly minor allele and low NE affinity AR were present but did not alter or even enhance efficacy in the presence of the major allele homozygous β_1_389 Arg genotype, which encodes ARs with a NE affinity of ∼100-fold more than 389 Gly ARs.

Combinatorial genotyping led to improvement in pharmacogenetic differentiation of drug response compared with monotype genotyping. The use of β_1_389 Arg/Gly and α_2C_322–325 Wt/Del genotype combinations accomplishes the goal of pharmacogenetics to identify response outliers from both ends of the therapeutic spectrum. Compared with the use of β_1_389 Arg/Gly or α_2C_322–325 Wt/Del monotypes, the differential efficacy gained by the use of genotype combinations was increased by respective amounts of 54% and 94%. The new identification of a completely unresponsive genotype, supported by biologic plausibility and bolstered by data consistency across multiple clinical endpoints, is especially important inasmuch as a major goal of pharmacogenetics is to identify patients with no likelihood of benefit who can then be spared drug side effects [21]. Other β-blockers that have been used to treat HF do not have these pharmacogenetic interactions [22], [23], but rather exhibit response heterogeneity through other, unknown mechanisms [8]. Thus, the ability to predict drug response through pre-treatment pharmacogenetic testing should improve therapeutic response to this drug class but will need to be confirmed by prospective studies.

Finally, the unexpected results of this study, (i.e., the additive loss of efficacy by minor allele combinations in the absence of additive gain of efficacy by major allele homozygotes) emphasizes that combinations of response-altering polymorphisms may behave in unpredictable ways and *in-silico* predictions of combinatorial genetic effects will need to be supported by empirical data.
